# Ingestion of mammalian meat and alpha-gal allergy: Clinical relevance in primary care

**DOI:** 10.4102/phcfm.v11i1.1901

**Published:** 2019-04-29

**Authors:** Tshegofatso Mabelane, Gboyega A. Ogunbanjo

**Affiliations:** 1 Allergy and Immunology Unit, University of Cape Town, Lung Institute, Mowbray, South Africa; 2 Department of Family Medicine and Primary Health Care, Sefako Makgatho Health Sciences University, Ga-Rankuwa, South Africa

## Abstract

**Background:**

An allergic reaction to mammalian meat has recently been reported in rural parts of South Africa and throughout other parts of the world. The cause of this allergic reaction is because of an oligosaccharide antigen known as galactose-alpha-1, 3-galactose (alpha-gal) found in mammalian meat. Hard ticks in various parts of the world have been identified as a cause of sensitisation to the alpha-gal antigen. However, mechanisms of sensitisation in Africa are poorly understood.

**Aim:**

The aim of this article is to review current literature on the alpha-gal allergy and mammalian meat ingestion and the family physician’s role in diagnosing and managing this condition.

**Method:**

Indexes were searched using the keywords in the following electronic databases: Elsevier Science Direct, Google Scholar, Medline and PubMed.

**Results:**

Clinical presentation of the alpha-gal allergy occurs typically as a delayed anaphylaxis occurring within 3–6 hours after the ingestion of mammalian meat. A subset of patients described in South Africa presented with a rapid onset of symptoms occurring within 45 minutes. Furthermore, some of these patients present with abdominal symptoms only, which may be mistaken as food poisoning. Diagnosis is based on a history of reaction to mammalian meats (especially to fatty portions or organs) and serum specific alpha-gal antibodies. The main management of the alpha-gal allergy is avoidance of red meat and in mild reactions treatment with oral H1 receptor antihistamines.

**Conclusion:**

Sensitisation to the alpha-gal allergy results in adverse reactions to red meat, with tolerance to turkey, chicken and fish. A family physician can safely manage this condition.

**Keywords:**

alpha-gal allergy; mammalian meat; management; primary care; specific IgE antibody; alpha-gal sensitisation.

## Introduction

An immune mediated immunoglobulin E (IgE) antibody response to a mammalian oligosaccharide epitope, galactose-alpha-1, 3-galactose (alpha-gal), is associated with severe reactions after ingestion of mammalian meat (beef, pork, lamb, venison, goat and bison). Patients with allergic reactions to red meat have been reported worldwide.

## Methods

The phrase ‘alpha-gal allergy’ along with ‘mammalian meat adverse reactions’ was initially used by the reviewers to search for articles. An article was included if it reported on a mammalian meat allergy with the clear distinction of other types of meat adverse reactions. Delayed reactions to mammalian meat were first described in 2009. Articles reporting on the alpha-gal antigen published between 1988 and 2017 in English were included in the search. Indexes in the following electronic databases were searched: Elsevier Science Direct, Google Scholar, Medline and PubMed.

## Review findings (results)

### Sensitisations to alpha-gal antigen

**FIGURE 1:**
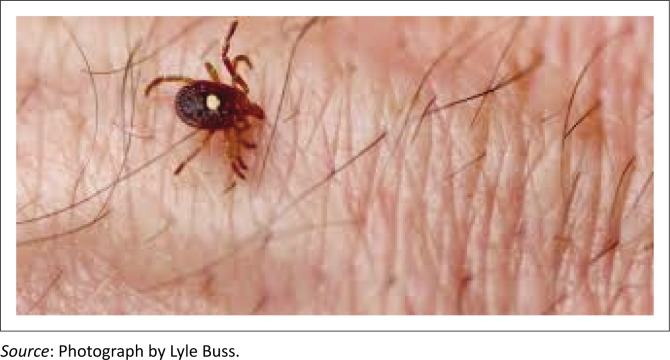
Lone star tick (*Amblyomma americanum*).^15^

Studies in Sweden and France have reported clear evidence that the causative ticks for alpha-gal sensitisation is *Ixodes ricinus*, while in Australia it is *Ixodes holocyclus* which is implicated for the allergy.^1,2,3,4^ In the United States, the predominant, if not exclusive, cause is the *Amblyomma americanum* (see Figure 1).^5^ There may be other associated causative factors for alpha-gal sensitisation. Oligosaccharides are well recognised as a target for antibody response to helminths, and it is known that helminths and ecto-parasites can give rise to IgE responses.^6,7^ However, there are no consistent IgE antibodies to alpha-gal in the serum of patients with documented helminth infections.^8^ The detection of anti-gal binding sites in some strains of *Escherichia coli, Klebsiella* and *Salmonella* suggests that this antigen could be present in the bacterial polysaccharides on the outer membranes of the bacterial flora in human intestines.^9,10^ Primary sensitisation to cat-serum albumin may result in the low-level detection of alpha-gal antibodies, not associated with a meat allergy.^11^ Alpha-gal sensitisation has been described in Harare, Zimbabwe,^12,13^ and Kenya^12,14^ and its association with the mammalian meat allergy in South Africa.^15^ However, no causative factors have been described.

### Alpha-gal in mammalian products

Alpha-gal is found in all mammalian cells but not in human and Old-World monkey tissues. Mammalian meat and milk (cow and goat) contain alpha-gal.^16^ Mammalian animal by-products may contain the alpha-gal epitope. However, animal by-products derived from turkey, chicken and fish do not contain the alpha-gal epitope.^17^

### Clinical presentations

**FIGURE 2:**
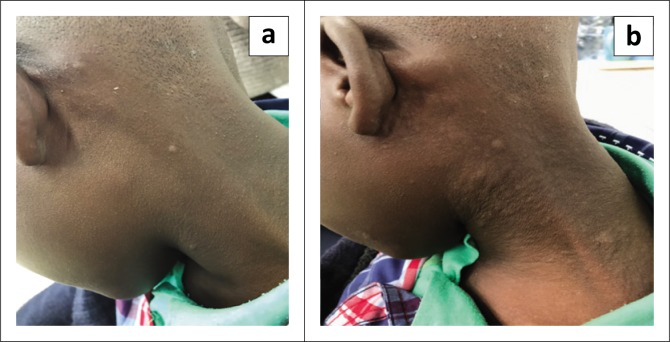
Progression of urticarial lesions during a reaction.

The alpha-gal allergy affects patients of all ages. Skin symptoms are commonly reported symptoms but may progress to anaphylaxis (see Figure 2).^18^ Research in the United States has shown that adult patients present with more severe reactions than children do. The onset of reactions is typically delayed and may result from eating meat 3–5 hours earlier.^16^ On the contrary, in South Africa, research conducted in the rural Eastern Cape showed a rapid onset of symptoms presenting with a high prevalence of gastrointestinal manifestation (abdominal pain, vomiting and diarrhoea). In some patients, sustained and severe abdominal pain was the only symptom without associated skin manifestations. In this cohort, severe symptoms were found to be more common in children than in adults.^19^

Reactions do not occur with every ingestion of red meat in comparison with a typical IgE-mediated food allergy where there is reproducibility of a reaction with every exposure to the trigger food. Patients mostly report reactions after eating large, fatty portions of red meat or organs, for example, kidney or liver.^16^ The rate of lipid absorption may play a role in the delayed reactions. Part of the delay may also be a result of conversion and processing of fats to chylomicrons and then further to low density lipoprotein particles of various sizes.^20^

### Diagnosis of alpha-gal allergy

The diagnosis of the alpha-gal allergy is based on a good medical history, a relevant blood test and oral food challenge (only if indicated).

#### Medical history

A detailed food allergy history focuses on symptoms, timing of symptoms in relation to ingestion, list foods ingested around the time of the reaction, the consistency of reactions upon exposure, how the food was prepared, whether additional factors were occurring at the time (exercise, illness, use of non-steroidal anti-inflammatory drugs), whether symptoms also occur without relationship to ingestion, treatments given and the time-course of the symptoms.^21^

A positive history of adverse reaction to mammalian meat or other mammalian products is suggestive of the alpha-gal allergy. Patients over the age of 5 years with an apparent new onset milk allergy should be investigated for the alpha-gal allergy.^21^

#### Blood test

A blood sample of at least 2 mL is collected in a plain tube using standard procedures and can be stored at 2 °C – 8 °C for up to 1 week and sent to a laboratory for testing. The test is a fluoro enzyme immunoassay, and this method detects antibodies against an allergen. A patient’s serum is coupled with the commercial alpha-gal antigen on a high binding capacity cellulose matrix. Any IgE that is specific for the antigen binds to the matrix. Anti-IgE is added to form a complex and make the antigen visible.^22^ Performing immunoassay testing for specific IgE to pork, beef and cat serum albumin is recommended to exclude other mammalian meat allergy conditions.^16^ There is a low titre of beef, pork or lamb sensitisation but high-specific IgE antibodies to alpha-gal. Sensitivity, specificity, negative predictive values and positive predictive values for alpha-gal IgE levels and alpha-gal: total IgE ratio, have been determined in the South African population.^19^

Patients with the alpha-gal allergy may have a positive blood test to cat IgA because of cross sensitisation.^23^

**FIGURE 3:**
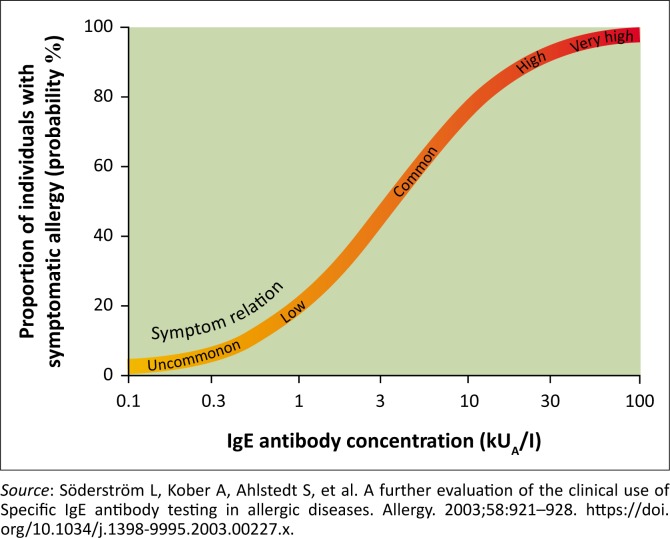
Specific IgE antibody levels and interpretation in symptomatic individuals.^44^

In clinical practice, specific IgE antibody levels of 0.35 kU/L are commonly considered as the cut-off point for sensitisation to an allergen. Low IgE antibody levels indicate a low probability of clinical disease, whereas high antibody levels to an allergen show a good correlation with clinical disease but not severity^24^ (see Figure 3).

Specific IgE antibodies are present in serum as a result of sensitisation and not necessarily allergy.

#### Oral food challenge

An open oral food challenge is performed in patients with alpha-gal antigen sensitisation but an unclear history of meat allergy. The principle of an oral food challenge is to give the suspected food in incremental doses at 15 minutes intervals, under close medical supervision.^25^ However, the alpha-gal allergy often presents with a delayed reaction and the oral food challenge is different from the traditional one. A full dose of 150 g of pork in an adult patient is given at once with close monitoring for at least 8 h. Informed consent is obtained before the procedure, and an experienced allergy clinician with resuscitation skills performs this procedure. Beef challenges produce more severe reactions than pork.^26^

### Differential diagnosis

#### Pork–cat syndrome

Pork–cat syndrome is an uncommon syndrome where patients develop an IgE antibody response specific for cat serum albumin that cross-reacts with porcine albumin. Patients classically present with a range of symptoms as in other food allergic reactions and the clinical symptoms are not predicted by the concentrations of specific IgE antibodies to cat serum albumin. The reaction is immediate and can be severe or even fatal upon consumption of pork. A subset of patients have presented with reactions to both pork and beef.^27,28,29^ Positive specific IgE to pork and cat serum albumin and negative specific IgE antibodies to alpha-gal confirm the diagnosis of the pork–cat syndrome. The natural history of the pork–cat syndrome is not well established, but it appears that the specific IgE to cat serum albumin may slowly decrease over time.^30^

#### Beef milk syndrome

Between 10% and 20% of children who are allergic to cows’ milk are also allergic to beef. Conversely, 93% of children allergic to beef are also allergic to milk. The molecular basis for this syndrome is allergy to bovine serum albumin.^31^ Industrial treatment, more than home cooking, may modify the allergic reactivity of this meat in beef-sensitive children, making industrially freeze-dried or homogenized beef safe alternatives to butcher’s meat cooked at home.^32^ Total avoidance of beef by children allergic to milk is not advised and needs an allergist’s evaluation.

### Management

#### Non-medical management

**Education:** The unpredictability of anaphylaxis may cause anxiety in patients and their families. A written action plan for the treatment of allergic reactions is useful in early identification of symptoms and appropriate management by patients and family members. Patients are instructed to wear bracelets that warn about alpha-gal hypersensitivity, for example, being allergic to red meat.^21^

**Dietary management:** The avoidance of red meat is the main management of the alpha-gal allergy.^21^ Patients react to red meat but usually tolerate other mammalian products. Clinical reactivity to all mammalian products results in complete avoidance strategies. An experienced dietitian with knowledge in the alpha-gal allergy will be the ideal person to guide a newly diagnosed patient with a mammalian meat allergy. Iron and vitamin B_12_ are supplements that are recommended to prevent deficiencies in patients with a long-standing requirement for avoiding mammalian meat.^33^

### Medical

#### Immediate management

Mild reactions such as urticaria or gastrointestinal symptoms are treated with oral H_1_ receptor antihistamines preferably in liquid form to enhance rapid absorption. However, there is evidence that other mediators such as platelet activating factors and kinins are associated with severe and potentially fatal reactions.^34,35^ The progression of symptoms is unpredictable, and in these patients, adrenalin must be considered if symptoms do not resolve.

The first line management for severe reactions is with undiluted adrenalin 1:1000 (1 mg/mL) 0.01 mL/kg (max 0.3 mL) in children and 0.5 mg intramuscularly (IMI) in adults administered on the anterolateral thigh. The adrenalin intramuscular injection results in a faster and better response, and a subcutaneous injection is not recommended. Another dose of adrenalin is repeated after 5 min if the patient did not respond. The patient is placed in a recumbent position. A call for help is necessary in managing patients with severe reactions. Patients on *β*-blockers are resistant to adrenalin and should receive glucagon 01.mg/kg (maximum 1 mg) every 20 min. Supportive treatment includes oxygen, bronchodilator and antihistamine. Corticosteroids are administered as second-line management to prevent a biphasic reaction. In hypotensive patients, resuscitation fluids are administered. Prolonged anaphylaxis requires extended observation and treatment.^36,37^

#### Long-term management

Adrenalin and a kit that includes syringes, needles, alcohol swabs and cotton are prescribed to patients with life-threatening reactions to mammalian meat. An epipen auto-injector is more convenient and can be prescribed as either Epipen Junior^®^ 0.15 mg or Epipen Senior^®^ 0.3 mg or according to the manufacturer’s specifications. Two sets of adrenalin are safe, especially in cases of a refractory reaction.^37^

#### Clinical implications of alpha-gal allergy

IgG antibodies against alpha-gal are naturally present in humans and constitute about 1% of circulating immunoglobulins in response to immune stimulation by enteric bacteria.^38^ Patients with the alpha-gal allergy produce IgE antibodies resulting in an allergic response.

#### Hypersensitivity reaction to antivenom

Deglycosylation assays and IgE inhibition tests confirmed that IgE-mediated reactivity to antivenom are associated with alpha-gal. Alpha-gal is a potential target of IgE-mediated reactivity to equine antivenom and a possible cause of the high incidence of hypersensitivity reactions during the first application of equine antivenom.^39^

#### Reactions to a prosthetic heart valve for aortic valve replacement

Mozzicato et al. reported three patients with suspected alpha-gal allergies, and of those, two developed immunologic reactions from porcine or bovine aortic valve replacement.^40^ Bio prosthesis was associated with the presence of alpha-gal in the valve tissue. The evidence of this description was strengthened by other groups and also proven in experimental animal work.^41,42,43^

### Prognosis

Specific IgE antibodies to alpha-gal appear to decrease over time. Patients with the alpha-gal allergy can have a resolution of symptoms and tolerate red meat again. However, symptoms re-occur with additional exposure to ticks. Follow-up is advised in these patients because of the possibility of reoccurrence after remission.

## Conclusion

The alpha-gal allergy should be considered in patients with idiopathic anaphylaxis or multiple potential causes for both acute and chronic urticaria, as well as angioedema.^21^ Patients with abdominal symptoms upon exposure to red meat may need to be assessed for the alpha-gal allergy. Further research is needed on the causative factors of the alpha-gal allergy in Africa and understanding the mechanisms of sensitisations for the implementation of preventative strategies. Diagnosis is based on a history of reaction to mammalian meats (especially to fatty portions or organs) and serum specific alpha-gal antibodies. The main management of the alpha-gal allergy is avoidance of red meat and in mild reactions treatment with oral H_1_ receptor antihistamines.
